# MRI Diagnosis of Gymnast’s Wrist (Distal Radial Physeal Stress Injury)

**DOI:** 10.5334/jbsr.4212

**Published:** 2026-03-13

**Authors:** Amal El Madani, Lokmane Taihi

**Affiliations:** 1 Department of radiology, Cliniques Universitaires Saint‑Luc, Brussels, Belgium

**Keywords:** gymnast’s wrist, distal radius, physis, magnetic resonance imaging, overuse injury

## Abstract

Gymnast’s wrist is an uncommon stress‑related injury of the distal radial physis caused by repetitive axial loading in skeletally immature athletes. We report the case of a 16‑year‑old elite gymnast presenting with progressive wrist pain without acute trauma. Magnetic resonance imaging demonstrated focal widening of the volar distal radial physis with adjacent metaphyseal and epiphyseal bone marrow edema, consistent with a stress‑related physeal injury. Conservative management with activity modification and physical therapy resulted in symptom resolution.

*Teaching point:* In young athletes with wrist pain, stress‑related physeal injury should be considered, and MRI allows early diagnosis and appropriate management.

## Introduction

Overuse injuries of the physis are increasingly encountered in skeletally immature athletes participating in high‑impact and weight‑bearing sports. Among these entities, gymnast’s wrist corresponds to an infrequent stress‑related injury of the distal radial physis induced by repetitive axial loading. Because early radiographic findings may be subtle or absent, magnetic resonance imaging (MRI) plays a pivotal role in early diagnosis and in preventing growth‑related complications. We report a typical MRI presentation of gymnast’s wrist in a young elite gymnast.

## Case Presentation

A 16‑year‑old male elite gymnast presented with progressive wrist pain evolving over several weeks. The pain developed insidiously, without any history of acute trauma, and was clearly correlated with training intensity, particularly during repetitive weight‑bearing activities on the hands.

Physical examination revealed localized tenderness over the distal radius, without swelling, deformity, or limitation of wrist motion. Neurovascular examination was normal.

**Figure 1:**
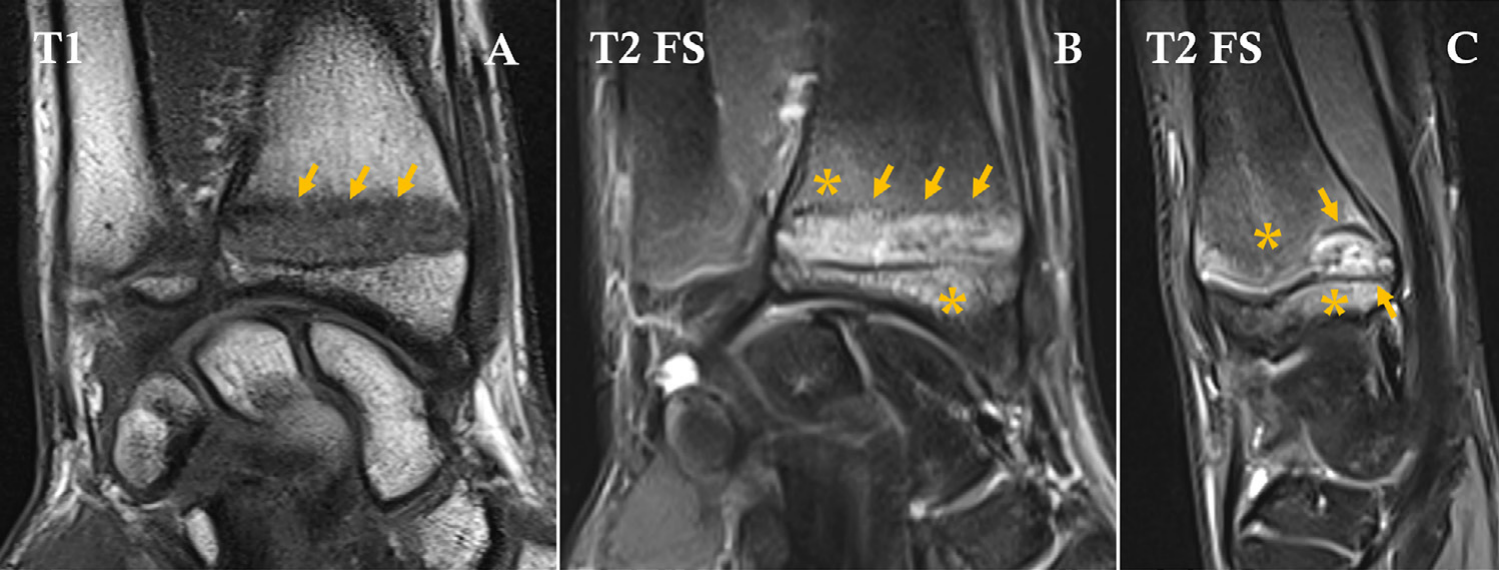
Coronal T1‑weighted **(A)**, coronal and sagittal T2‑weighted fat‑suppressed **(B, C)** MR images demonstrating focal widening of the volar distal radial physis (arrows) with adjacent metaphyseal and epiphyseal bone marrow edema (asterisks).

MRI of the wrist demonstrated focal widening of the volar portion of the distal radial physis with irregular physeal margins, showing high signal intensity on fluid‑sensitive sequences, associated with adjacent metaphyseal and epiphyseal bone marrow edema (Figure 1). No fracture line, cartilage disruption, or signs of premature physeal closure were identified. The surrounding soft tissues, including tendons, ligaments, and the triangular fibrocartilage complex (TFCC), were unremarkable.

Based on the clinical context and imaging findings, a diagnosis of stress‑related distal radial physeal injury, consistent with gymnast’s wrist, was established. Conservative management with temporary avoidance of axial loading, training adaptation, and supervised physical therapy led to progressive symptom resolution.

## Discussion

Gymnast’s wrist is an overuse injury caused by repetitive compressive forces across the immature distal radial physis. It predominantly affects skeletally immature athletes involved in sports requiring frequent wrist loading, such as gymnastics and weightlifting. During growth, the physis represents a biomechanically weak component of the bone and is particularly vulnerable to repetitive microtrauma.

Chronic axial loading results in physeal stress, leading to widening and irregularity of the growth plate, often accompanied by reactive metaphyseal bone marrow edema. These MRI findings reflect an imbalance between mechanical load and the adaptive capacity of the developing physis rather than an acute traumatic process.

MRI is the imaging modality of choice for early diagnosis. Conventional radiographs may appear normal in early stages or show subtle physeal widening only in advanced cases. MRI allows direct visualization of the physis and adjacent bone marrow and is highly sensitive for detecting stress‑related changes. Typical MRI features include metaphyseal bone marrow edema on fluid‑sensitive fat‑suppressed sequences, associated with focal or diffuse physeal widening and irregular physeal contours. MRI also enables comprehensive evaluation of surrounding soft tissues and detection of complications [1].

Early recognition is essential, as continued mechanical stress may result in premature closure of the distal radial physis. This growth disturbance can lead to positive ulnar variance—predisposing patients to secondary ulnocarpal impaction syndrome and TFCC injury—or to Madelung‑type deformity, potentially resulting in long‑term functional impairment [2].

The differential diagnosis of wrist pain in young athletes is broad. Clinically, stress fractures of the carpal bones, as well as ulnocarpal impaction syndrome, TFCC injuries, tendinopathies, and tenosynovitis can be considered. From an imaging perspective, physeal‑centered abnormalities may have several causes. Distal radial Salter–Harris fractures can mimic gymnast’s wrist but are typically associated with acute trauma and demonstrate sharp physeal disruption rather than diffuse widening with irregular margins. Focal periphyseal edema (FOPE) zone represents a usually asymptomatic physiological phenomenon related to focal physeal closure in late adolescence and is characterized by physeal‑centered edema with focal physeal narrowing rather than widening. Infectious conditions, such as septic osteomyelitis or chronic recurrent multifocal osteomyelitis, may also involve the physis but are associated with a distinct clinical and biological context.

Management of gymnast’s wrist is primarily conservative and includes rest, activity modification, and, when necessary, temporary immobilization. Prognosis is generally favorable when diagnosed early and when mechanical stress is adequately reduced.

## Conclusion

Gymnast’s wrist is an important and potentially underrecognized cause of chronic wrist pain in skeletally immature athletes. MRI plays a central role in early diagnosis, assessment of disease severity, and exclusion of differential diagnoses. Prompt recognition and appropriate management are essential to prevent irreversible growth disturbances and long‑term complications.
